# In Silico Analysis and Functional Characterization of Antimicrobial and Insecticidal Vicilin from Moth Bean (*Vigna aconitifolia* (Jacq.) Marechal) Seeds

**DOI:** 10.3390/molecules27103251

**Published:** 2022-05-19

**Authors:** Muhammad Ateeq, Muhammad Muzammal Adeel, Ayesha Kanwal, Muhammad Tahir ul Qamar, Ahsan Saeed, Binish Khaliq, Qamar Saeed, Muhammad Nauman Atiq, Muhammad Bilal, Metab Alharbi, Abdulrahman Alshammari, Ahmed Akrem

**Affiliations:** 1 Key Laboratory of Horticultural Plant Biology-Ministry of Education, College of Horticulture and Forestry Sciences, Huazhong Agricultural University, Wuhan 430070, China; ateeqmuhammad21@gmail.com; 2 Botany Division, Institute of Pure and Applied Biology, Bahauddin Zakariya University, Multan 60800, Pakistan; ahsansaeed179@gmail.com; 3 Hubei Provincial Key Laboratory of Agricultural Bioinformatics, College of Informatics, Huazhong Agricultural University, Wuhan 430070, China; m.muzammal.adeel@outlook.com; 4 Department of Environmental Health Science, College of Public Health, University of Georgia, Athens, GA 30602, USA; 5 College of Life Sciences, University of Science and Technology of China, Hefei 230027, China; ayesha_comsian@hotmail.com; 6 Department of Bioinformatics and Biotechnology, Government College University, Faisalabad 38000, Pakistan; 7 Department of Botany, Faculty of Life Science, University of Okara, Okara 56300, Pakistan; binish@uo.edu.pk; 8 Department of Entomology, Bahauddin Zakariya University, Multan 60800, Pakistan; qamarsaeed@bzu.edu.pk; 9 Hubei Insect Resources Utilization and Sustainable Pest Management Key Laboratory, College of Plant Science and Technology, Huazhong Agricultural University, Wuhan 430070, China; mian.nauman911@gmail.com; 10 Centre of Excellence in Molecular Biology, University of the Punjab, 87-West Canal Road Thokar Niaz Baig, Lahore 53700, Pakistan; bilal.camb@pu.edu.pk; 11 Department of Pharmacology and Toxicology, College of Pharmacy, King Saud University, P. O. Box 2455, Riyadh 11451, Saudi Arabia; tahirulqamar@gcuf.edu.pk (M.T.u.Q.); Abdalshammari@ksu.edu.sa (A.A.)

**Keywords:** moth bean, biomolecules, vicilin, structure prediction, antimicrobial, insecticidal

## Abstract

Vicilin has nutraceutical potential and different noteworthy medicative health-promoting biotic diversions, and it is remarkable against pathogenic microorganisms and insects. In this study, *Vigna aconitifolia* vicilin (*Vac*V) has been identified and characterized from the seed of *Vigna aconitifolia* (Jacq.) Marechal (Moth beans). LC-MS/MS analysis of *Vac*V provided seven random fragmented sequences comprising 238 residues, showing significant homology with already reported *Vigna radiata* vicilin (*Vra*V). *Vac*V was purified using ammonium sulfate precipitation (60%) followed by size exclusion chromatography on Hi-Load 16/60 Superdex 200 pg column and anion-exchange chromatography (Hi trap Q FF column). Purified *Vac*V showed a major ~50 kDa band and multiple lower bands on 12% sodium dodecyl sulfate polyacrylamide gel electrophoresis (SDS-PAGE) under both reduced and non-reduced conditions. After all, a three-dimensional molecular structure of *Vac*V was predicted, which showed β-sheeted molecular conformation similar to crystallographic structure of *Vra*V. All Vicilins from *V*. *aconitifolia* and other plants were divided into six sub-groups by phylogenetic analysis, and *Vac*V shared a high degree of similarity with vicilins of *Vigna radiata*, *Pisum sativum*, *Lupinus albus*, *Cicer arietinum* and *Glycine max*. Additionally, *Vac*V (20 μg) has significant growth inhibition against different pathogenic bacteria along strong antifungal activity (50 μg). Likewise, *Vac*V (3.0 mg) produced significant growth reduction in Rice Weevil *Sitophilus oryzae* larvae after 9 days compared with control. Furthermore, by using MMT assay, the cytotoxicity effect of *Vac*V on the growth of HepG2 liver cancerous cells was tested. *Vac*V showed cytotoxicity against the HepG-2 line and the acquired value was 180 µg after 48 h. Finally, we performed molecular docking against caspase-3 protein (PDB ID: 3DEI) for *Vac*V bioactive receptor interface residues. Hence, our results reveal that *Vac*V, has nutraceutical potential and moth beans can be used as a rich resource of functional foods.

## 1. Introduction

Legumes (pulses) are widely grown and a good source of nutrients. Their dietary and economic importance is globally recognized. Total production of legumes has been increasing for decades in Europe and worldwide, ~34% and 44%, respectively [1]. Moth bean is an annual pulse that belongs to the family *Fabaceae*, commonly cultivated in the tropical-arid and semi tropical-arid zones of Africa, Latin America, and South Asia, especially India and Pakistan [2,3]. Moth bean seeds are used as a food rich in carbohydrates, proteins, fats, vitamins and minerals [4,5,6]. Dietary compounds/ingredients of moth bean have become extremely relevant in recent studies [7,8].

Seed storage proteins (SSPs) largely found in pulses and beans are often used as food elements and impart certain features such as plant growth, development, and defense during seed germination. SSPs are synthesized in membrane-bounded organelle such as rough endoplasmic reticulum (RER) [9,10]. Albumins, globulins, prolamin, and glutelin are the major SSPs found in the moth bean [11]. Vicilins (legume lectin) are 7S globulins found in abundance as reserves in seeds of leguminous and non-leguminous plants, representing as much as 70 to 80% of total protein in *Vigna unguiculata*, *Vigna radiata*, *Glycine max*, *Phaseolus vulgaris*, *Canavalia ensiformis*, *Lupinus albus* and *Vigna aconitifolia* [12,13]. Vicilin is characterized as trimeric oligomers of 150–170 kDa formed by three similar subunits of 40–70 kDa with no disulfide linkages [14]. The individual subunits are *N*-glycosylated low-complexity regions and are composed of a core with loop domains, called cupin superfamily domains (conserved domains) in the C-terminal [15,16]. The conserved and functionally diverse cupin domain superfamily was previously reported in prokaryotes and eukaryotes [9]. The N-terminus of vicilin and 7S globulins is designed with 50–60 amino acids that contain antimicrobial motifs [9,15,16,17,18].

Antimicrobial and insecticidal activities of vicilins from *V*. *unguiculata*, *G*. *max*, *V*. *radiata*, *C*. *ensiformis*, *P*. *vulgaris*, *L*. *albus*, *M*. *integrifolia* and *P*. *sativum* have been investigated [19,20,21]. However, no attention has been paid to the vicilin of *V. aconitifolia*. Microbes (virus, bacteria and fungi) and insects may cause many problems that have an effect on human life and the shelf life of food [22,23]. Uneven utilization and overuse of antimicrobial drugs have induced antibiotic-resistance bacteria and researchers have focused on investigating new antimicrobial agents with different mechanisms of action to be used as food preservatives or in human diseases treatment [21]. Similarities of vicilin protein components between *V*. *radiata* and other legumes propose similar functions and applications [24,25]. The current research focuses on in silico structural and in vivo functional characterization of *V*. *aconitifolia* seed “vicilin”. Hence, plant-based chemicals (proteins) with biological activity are a possible alternative to conventional medications and antibiotics.

## 2. Results

### 2.1. Purification of VacV

**Figure 1:**
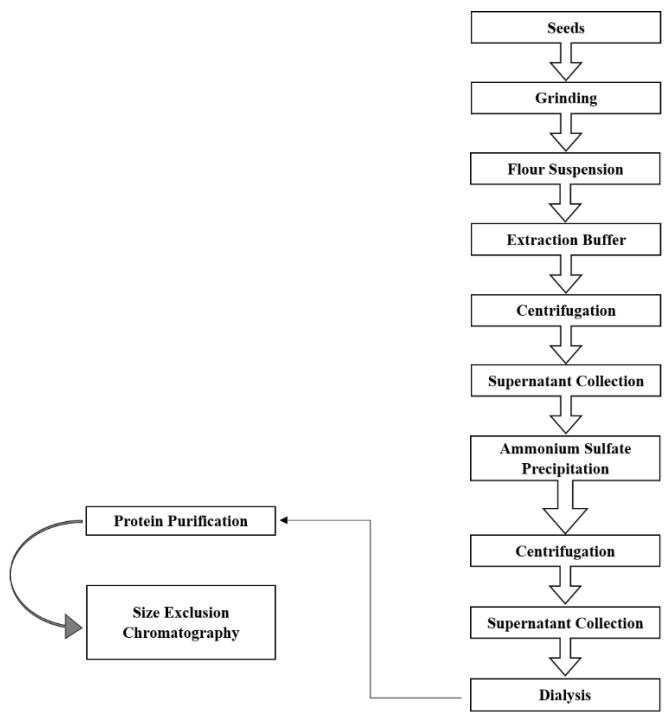
Schematic diagrams for protein purification stages.

**Figure 2:**
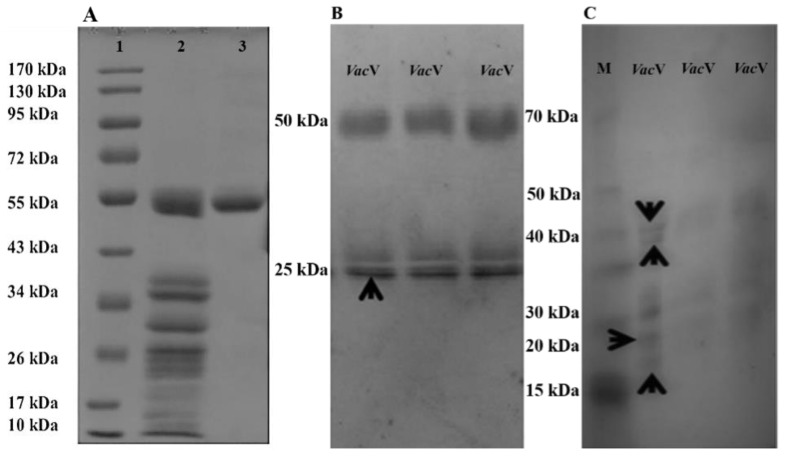
SDS-PAGE analysis of purified *V*. *aconitifolia* vicilin (*Vac*V) protein under reduced and non-reduced conditions. (**A**) Lane 1 (marker Catalog no. BM201), *Vac*V crude extract (lane 2) and highly purified *Vac*V band (lane 3). (**B**) 50 kDa *Vac*V mother band and the corresponding lower range (~25 kDa) bands, which are actually the cleaved products of 50 kDa mother band. (**C**) Gel is showing the further cleavage of 50 kDa mother band after seven days of storage at 4 °C. Lane M is loaded with a standard protein marker. Lanes *Vac*V are showing the cleaved lower molecular weight bands. In (**A**–**C**), black arrows are indicating the bands excised for amino acid sequencing through mass spectrometry.

Extraction, purification fold and recovery percentage of *Vac*V have been shown in Table 1 and Figure 1. Briefly, one gram of seeds yielded over 350 mg of crude extract protein in phosphate buffer (100 mM; pH 7.0), which was reduced to 122.5 mg yield followed by anion and gel filtration chromatography (2.85 times purified). Finally, gel filtration continued by anion exchange chromatography helped in further purification of the protein (homogeneity grade) as given in Figure S1A–D, with a 3.48% yield of *Vac*V (28.6 times purified). SDS-PAGE showed a single band of 50 kDa detected under both reduced and non-reduced conditions (Figure 2A). However, the 50 kDa mother band was completely removed and proteolytic lower bands appeared after a few days of storage (Figure 2B,C). 

### 2.2. Protein Identification by LC-MS/MS Spectrometry

**Figure 3:**
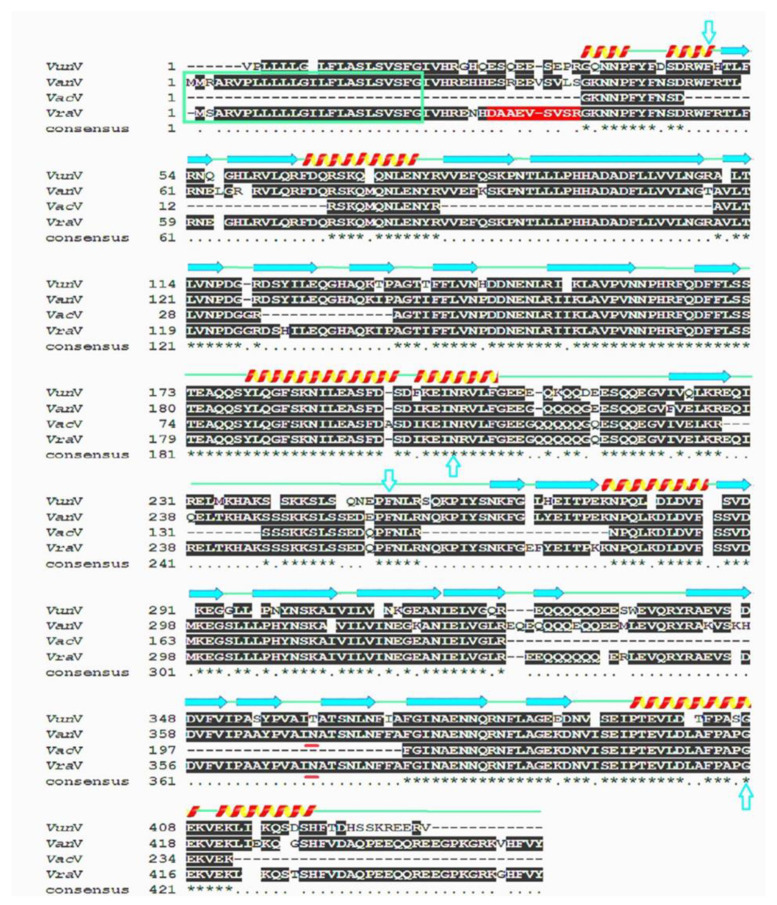
Multiple sequence alignment and evolutionary assessment of *Vac*V. Multiple sequence alignment of *Vac*V with *Vigna unguiculata* (*Vun*V), *Vigna angularis* (*Van*V), and *Vigna radiata* (*Vra*V). Identical amino acids of four vicilin proteins indicated by asterisks. Arctic arrows, green lines and red and yellow coiled structures showing alpha helices, β-strands and conserved regions, respectively.

Purified *Vac*V was exposed to overnight tryptic digestion and fragments were loaded in LC-MS/MS spectrometer. Excised gel bands of *Vac*V (50 kDa) (Figure 2B) produced seven random fragments that yielded 238 residues (Table 2). The tryptic peptide sequence, GKNNPFYFNSDR, was BLASTed in the UniProtKB online server3 [26,27] and found to be 100% identical to previously reported vicilins from *V*. *radiata*, *P*. *vulgaris*, and other plants (Table 3). Fragmented residual sequences were used for multiple sequence alignment with vicilins of *V. angularis*, *V. radiata*, and *V. unguiculata* (Figure 3). Alignment analysis indicated that *Vac*V is more identical to *V. radiata* vicilin. *V. radiata* vicilin (*Vra*V) is composed of two conserved Cupin domains (Cupin-I, 54–206; Cupin-II, 259–419) separated by a stretch of almost 52 residues, and residual data of *Vac*V is dispersed all over the two domains. Identical, similar and dissimilar amino-acid residues among the four proteins are indicated by asterisks, dots, and gaps, respectively. α-helices are indicated by spirals and β-strands by arrows; the green box shows the signal peptide sequence; the red box shows the propeptide sequence; N-terminus is shown in the red background; turquoise arrows indicate the Cupin I (54–206) and Cupin II (259–419) domains of vicilin, respectively. Each Cupin domain is further composed of a 6-stranded beta-barrel structure. The lime box is indicating one potential nick for proteolytic cleavage. The single vicilin glycosylation site has been marked with a yellow box (N-A-T) with asparagine being the probable residue for linking of carbohydrate moieties. Since all four sequences are totally devoid of cysteine residues, inter or intra-chain disulfide linkages are absent. Conserved domains play an important role in the regulatory functions of vicilins [15,16].

### 2.3. VacV Structure Prediction and Phylogenetic Analysis

**Figure 4:**
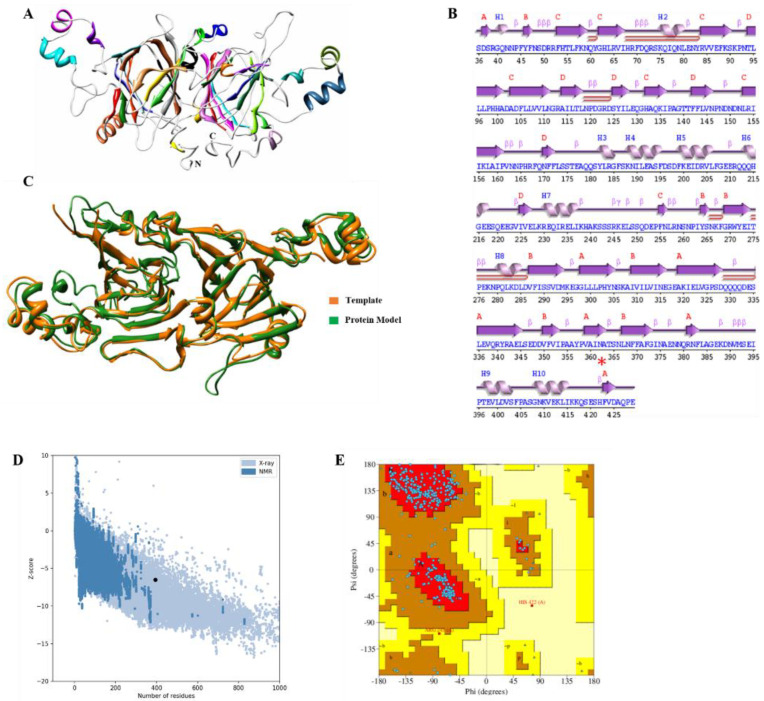
3D-structure in *Vac*V protein. (**A**) Ribbon representation of Vicilin protein three-dimensional model, different components of protein such as helices, sheets and loops are highlighted with different colors. N-terminus and C-terminus of protein are shown with N and C, respectively. (**B**) Secondary structure of *Vac*V protein. (**C**) Superimposition of vicilin template and globulin protein, Green; Vicilin protein model, and gold globulin protein model. (**D**) Z-score calculation to estimate the quality of model; black dot representing the position of predicted model. (**E**) Ramachandran plot; blue dots showing amino acid residues. Upper left and lower left panel belong to allowed regions, while upper right and lower right are the disallowed regions.

The computational three-dimensional protein structure of *Vac*V showed a very stable arrangement of amino acids. Structural quality analysis suggested that the predicted model is of “good” quality, assisted by a high z-score, i.e., −6.5 (Figure 4A). Protein conformational analysis predicted that *Vac*V has four sheets, three “antiparallel” and one “mixed,” six beta hairpins, ten beta bulges, 27 strands, ten helices, 44 beta turns, and three gamma turns (Figure S2), only 1.41% of residues were detected as poor rotamers, 94.39% of residues belong to the Ramachandran favorable region, and no bad angles or bonds were detected (Tables S1–S9). Moreover, the structure also showed five helix–helix interactions between helix 1–2, helix 2–3, helix 4–3, and helix 3–5, as shown in Figure 4B. *Vac*V structural superimposition with the template showed high similarity with RMSD value of 0.84 Å (Figure 4C–E). 

**Figure 5:**
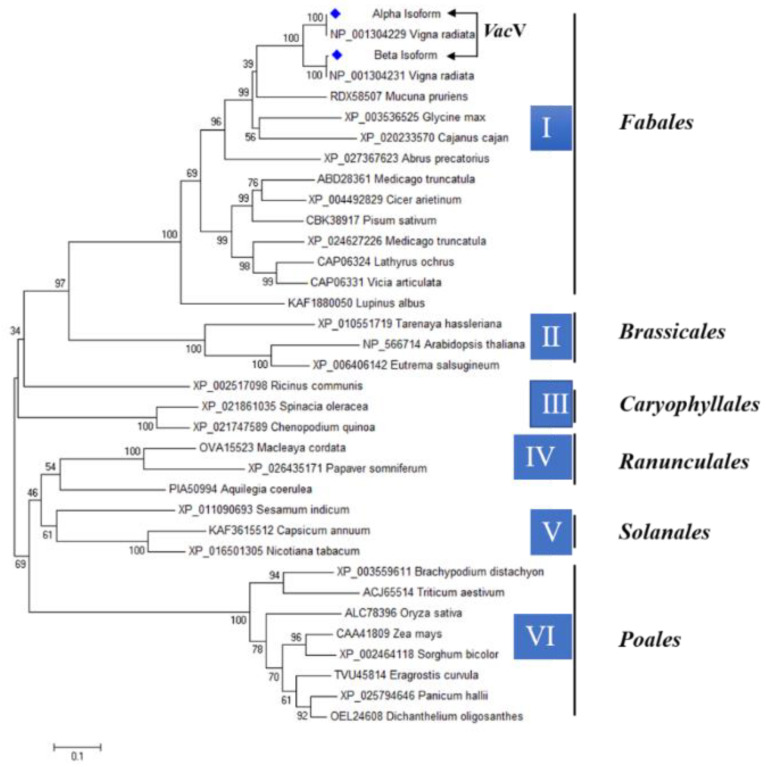
Phylogenetic trees of different plants based on vicilin protein. The tree was constructed by with neighbor joining method for 1000 bootstrap values, branch lengths are proportional to the number of substitutions per site with 0.10 scale bar. Blue diamante shape is showing *vigna aconitifolia* vicilin (*Vac*V) 100% similar with vicilin of *Vigna radiata*, member of *Fabales* order.

To investigate the phylogenetic relationship between *Vac*V and vicilin of different plant species, a phylogenetic tree was created based on the alignment of their full amino acid sequences. Based on similar taxonomic orders, the phylogenetic tree revealed that vicilin protein in different plants could be divided into six major groups (designated I, II, III, IV, V, and VI). Maximum similarity was shown between different species categorized according to *Fabales*, *Brassicales*, *Caryophyllales*, *Ranunculales*, *Solanales*, and *Poales* orders. When compared to the *Poales* order, *vicilin* protein shows less evolution and more similarity in the *Fabales* order. There was 100% vicilin protein similarity in *vigna aconitifolia* and *Vigna radiata*, which showed that there is evolutionary and structural identical in the synthesis of vicilin protein, which sharing the same genes of vicilin protein with Cupin-superfamily as given in Figure 5. 

### 2.4. Antibacterial Activity Assay

**Figure 6:**
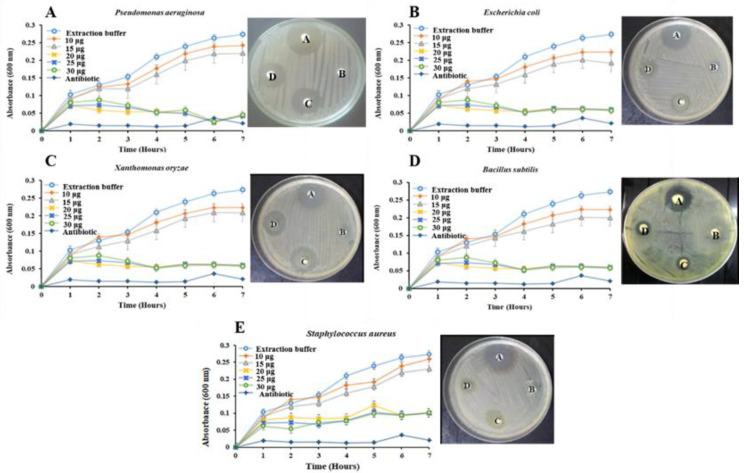
Effect of *Vac*V on the growth of pathogenic bacterial strain. Disc A is a positive control using Amoxicillin (1 mg ml^−1^). Disc B and C were indicating two different concentrations of *Vac*V as 15 and 20 µg/disc, respectively. Disc D is 30 μL buffer (100 mM Phosphate, pH 7.5) as a negative control. Clear inhibition zones were observed towards higher concentrations of *Vac*V against different bacterial growth. (**A**) *Pseudomonas aeruginosa*, (**B**) *Escherichia coli*, (**C**) *Xanthomonas oryzae*, (**D**) *Bacillus subtilis* and (**E**) *Staphylococcus aureus*.

The *Vac*V antibacterial activity was tested against five (*Staphylococcus aureus*, *Escherichia coli*, *Bacillus subtilis*, *Pseudomonas aeruginosa* and *Xanthomonas oryzae*) human pathogenic bacterial species. The levels of bacterial growth inhibition were different at different *Vac*V concentrations. The lower concentration of *Vac*V (10 µg) did not produce any significant growth inhibition of pathogenic bacteria in comparison to control. However, *Vac*V strongly inhibited the growth of both Gram-positive and Gram-negative bacteria at a concentration of 15 and 20 µg. A significant decrease in OD (at 600 nm) of bacterial growth was observed at a 20-µg dose with a little further decrease in the remaining concentrations of 25 and 30 µg. Further, two growth-inhibiting concentrations (0.6 µM and 1.2 µM), as depicted by Microtiter assay, were tested against bacterial strains by using the disc diffusion method. The two *Vac*V concentrations produced strong zones of inhibition against all pathogens, while no activity was observed around discs soaked with PBS buffer (Figure 6. Overall, *Vac*V has a potent ability to fight against *P. aeruginosa*, *E. coli*, *X. oryzae*, *B. subtilis*, and *S. aureus*. *Vac*V has significant antibacterial activity against *P. aeruginosa* and *X. oryzae* on 15 μg/disc (diluted) and 20 μg/disc (concentrated) concentrations (Figure 6A,C), while *Vac*V has the least significant effect on the same concentration growth inhibition zone against *B. subtilis*, *E. coli* and *S. aureus* (Figure 6B,D,E). The diameter of the inhibition zone (21 ± 1 mm) produced by *Vac*V was seen in the case of *X*. *oryzae* (20 µg/disc) followed by *P*. *aeruginosa*, *B*. *subtilis*, *E*. *coli* and *S*. *aureus* with inhibition zones 20 ± 3, 18 ± 2, 17 ± 2 and 16 ± 2 mm, respectively. The antimicrobial activity and antibiotic inhibition zones were measured as mean values of three replicates, as presented in Table 4.

### 2.5. Antifungal Assessment Activity

**Figure 7:**
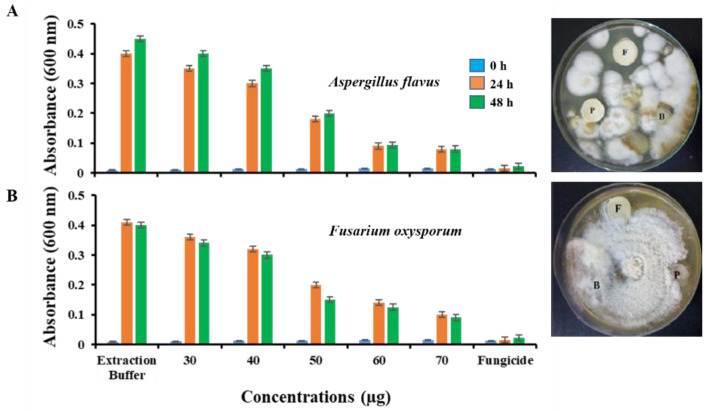
Antifungal activity of *Vac*V against fungal pathogens. (**A**) *A. flavus* and (**B**) *F. oxysporum*. A time scale study of fungal growth against different *Vac*V treatments indicated maximum inhibition at 2.5 µM concentration with little improvement beyond this value. It was also observed that in the first 24 h of *Vac*V exposure produced more than 50% of conidial germination inhibition, which remains constant. Disc F was used as a positive control containing fungicide (TOPSIN^®^ 10 µL/disc). Disc B was PBS 100 mM, pH 7.5 (50 µL/disc) used as a negative control. Disc P is indicating the *Vac*V concentration of 50µg/disc.

The *Vac*V antifungal activity was tested against *Aspergillus flavus* and *Fusarium oxysporum* pathogenic fungal species. After 48 h of incubation, conidial germination and mycelial growth in response to different *Vac*V concentrations were detected. As shown in Figure 7, the good antifungal activity of *Vac*V was observed against *A. flavus* and *F. oxysporum*. *Vac*V concentration of 50 µg/well showed more than 50% inhibition of the mycelia growth of fungal strains after 24 h of treatment with significant improvement of inhibition for 60 and 70 µg/well concentrations in the Microtiter assay. The disc diffusion assay also supported the data by displaying a similar pattern of growth inhibition at 50 µg/disc after 48 h of incubation compared with PBS (no inhibitory effect on fungal growth). It was also observed that *A. flavus* was more vulnerable to *Vac*V in comparison to *F. oxysporum* (Figure 7A,B). Fungal growth inhibition data is confirming the strong potency of *Vac*V against phytopathogenic fungi.

### 2.6. Determination of Insecticidal Activity

**Figure 8:**
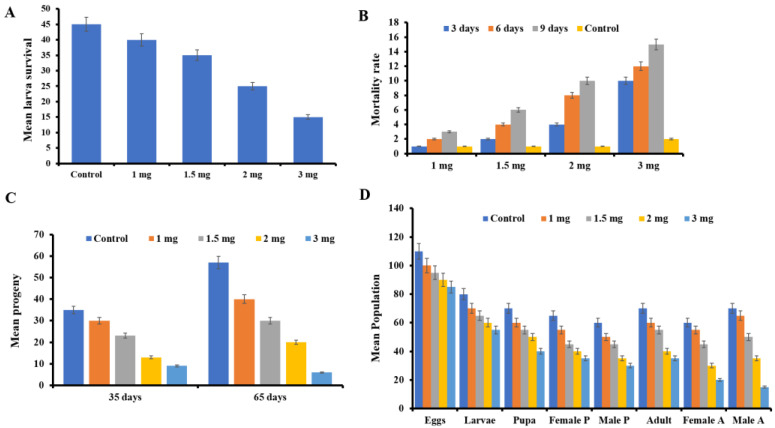
*Vac*V cytotoxicity against the *S. oryzae* (**A**) Mean larvae survival (±SE) of *S*. *oryzae* adults in response to *Vac*V protein after nine days of treatment. (**B**) *S*. *oryzae* mean percent mortality (±SE) after four treatments of *Vac*V sprayed on rice kernels. In comparison to each other and to the control, two *Vac*V treatments (1.5, 2.0, and 3.0 mg) resulted in considerable mortality. However, there was no significant mortality with the 1.0 mg therapy. After every third day, up to a maximum of nine days, data was collected until the adults mated and laid the eggs. (**C**) After 35 days, *Vac*V protein effectiveness against *S*. *oryzae* offspring. Purified *Vac*V has a considerable effect on growing larvae, as shown in the graph. A maximum protein concentration of 3.0 mg resulted in a significant reduction in developing larvae. (**D**) In response to *Vac*V, two generations of *S. oryzae* offspring were investigated. After 35 (first life cycle; F1) and 65 (second life cycle; F2) days, the effect of *Vac*V on two generations (F1 and F2) of *S*. *oryzae* offspring was observed. In comparison to the control, *Vac*V caused considerable mortality in the F1 generation; however, the F2 generation demonstrated less mortality in the adults. Nonetheless, when compared to the other therapies, the highest dosage (3.0 mg) resulted in considerable mortality.

Besides antimicrobial activity, *Vac*V also has potency against the larvae of *Sitophilus oryzae*. Adults of the parent *S*. *oryzae* generation experienced significant mean percent mortality in response to two *Vac*V treatments (2.0 and 3.0 mg/20 g of rice kernels per/replicate) after 3, 6, and 9 days of observation as compared to controls, although the lowest concentration of 1.0 mg had the least effect (Figure 8A,B). However, compared to the control group, 3.0 mg treatment resulted in a maximum mean mortality of 15.46 ± 2.5 after 9 days. Similarly, interesting inhibitory results were observed in the case of hatching progeny of *S. oryzae* from egg to pupa and it was observed that maximum mortality took place at the larval stage (3.0 mg). 

Adults for all four treatments were counted after a complete life cycle of *S*. *oryzae* F1 generation (35 days). In comparison to the control group, three *Vac*V treatments (1.5, 2.0 and 3.0 mg) resulted in a considerable reduction in the adult population. Furthermore, the maximum dose of 3.0 mg was shown to be statistically significant when compared to the other doses, which were not significant. After 65 days in the second generation (F2), the highest protein dose (3.0 mg), least rice weevils were observed on highest protein dose, i.e., 6.54 ± 0.55 followed by 2.0 and 1.5 mg as 20.33 ± 2.90 and 30.67 ± 1.76, respectively. As shown in Figure 8C, rice weevil progeny was the highest in the control group (60.23 ± 4.04). On all four doses of *Vac*V, the F2 generation demonstrated lower mortality than the F1 generation. Results clearly indicated the insecticidal activity of all four doses, which reduced the number of *S. oryzae* larvae with comparison to control-treated with buffer only.

### 2.7. Effect of VacV on Life Stage of S. oryzae

In addition, *Vac*V had a significant effect on different life stages of *S. oryzae*. *Vac*V, at a concentration of 3.0 mg/mL, significantly reduced the mean number of *S. oryzae* eggs with a population number of 90.7 ± 5.8 in comparison to the control population of 110.3 ± 9.3. Similarly, the larval population was observed to be 56.4 ± 4.8 at the 3.0 mg level dose, 62.8 ± 3.3 at the 2.0 mg concentration and 75.4 ± 5.6 at 1 mg. The highest mean number of larvae was observed in the control group (84.6 ± 7.6). When compared to the control group (78.28.59), larvae that survived and transformed into pupa stage showed a maximum reduction in number, i.e., 40.63.67 at a maximum dose of 3.0 mg/mL. Male and female pupae numbers were also recorded (35 ± 3.84 F, 30 ± 2.84 M), (39.6 ± 3.10 F, 27.6 ± 3.10 M), and (60.8 ± 4.56 F, 55.2 ± 4.46 M) for three treatments (3.0, 2.0, 1.0 mg/mL, respectively). Similarly, a significant declivity was detected in the mean population number of adults at the maximum dose of 3.0 mg (35.3 ± 3.7), followed by 2.0 mg (45± 4.7). A significant variance was observed in the mean number of males and females at the 3.0 mg/mL dose (20.1 ± 1.5 F) and 15 ± 2 M). However, the highest numbers of *S. oryzae* were observed throughout all stages in the control group, as shown in Figure 8D.

### 2.8. Evaluation of Cell Cytotoxicity

**Figure 9:**
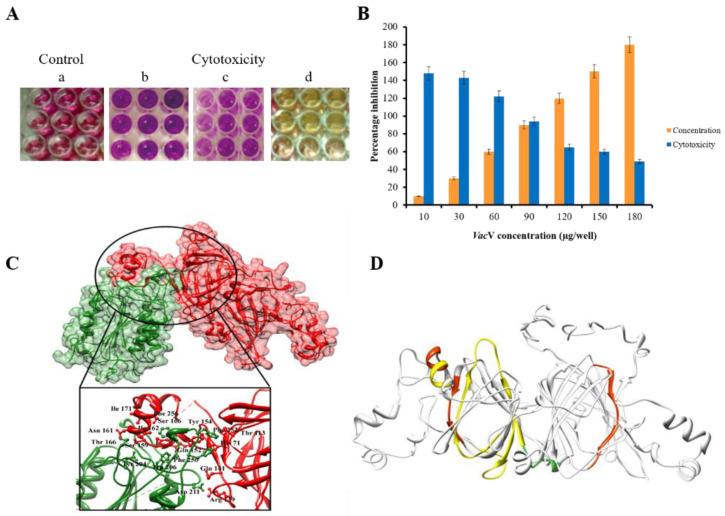
(**A**) The MTT assay was performed on a 96-well microplate; (a) control wells with untreated cells, (b) cell lines with 120 μg/well of *Vac*V protein, (c) cell lines with 150 μg/well of *Vac*V protein and (d) cell lines with 180 μg/well of *Vac*V protein (**B**) *Vac*V cytotoxicity towards HepG-2 cancerous lines according to MMT assay. Different concentrations of the protein used for a period of 48 h. Lower concentration actually produced non-significant result in comparison with control. However, higher concentration of 180 µg per well produced about 70% of the cell growth inhibition. (**C**) Superimposition of *Vac*V (red) and Caspase-3 (green) proteins. Upper penal showing surface view of dock complex and lower penal (magnified) showing interacting residues which involved in activity of vicilin protein. Interaction residues were refined on the base of bond length threshold <3 Å. (**D**) Bioactive active peptides are visualized with different colors: Yellow: peptides showing antioxidant activity; Green: peptides involved in antifungal activity and insecticidal activity; Deep red: peptides with antibacterial activity.

Purified *Vac*V has been tested against the HepG-2 liver cancerous cell lines. MMT assay was carried out using different concentrations (10, 30, 60, 90, 120, 150 and 180 µg) of *Vac*V per well (Figure S3). Cytotoxicity was evaluated for a total period of 72 h and after every 24 h, optical densities were recorded of the incubation at 570 and 630 nm. There was no cytotoxic effect experienced at initial concentrations of the *Vac*V as compared to control, IC50 was experienced at 180 µL of *Vac*V after 48 h as shown in Figure 9A,B. Caspase-3 have been reported as the potential anticancerous agent activator by binding with the anticancerous proteins [28]. Thus, it is worthy to explore its binding pattern with the *Vac*V. Molecular docking results suggested the presence of several hydrogen interactions with the receptor *Vac*V and the docking score—247.44 [29]. Interaction residues and bioactive peptides were refined on the base of bond length threshold <3 Å as shown in Figure 9C,D. Detailed information of interacting residues is given in Table S10. 

## 3. Discussion

In the South Asia region, legumes are extensively ingested, and an expanding body of clinical evidence shows significant immunological cross-reactivity [30]. In legume seeds, vicilins have a high degree of sequence homology and micro-diversity, suggesting that they may play a significant role in plant defense mechanisms and act as antimicrobial agents [31]. Vicilin belongs to the 7S globulin class, usually have a high molecular mass with no disulfide bonds and are present in the seeds of leguminous and other plants [32,33]. It inhibits the growth of human bacterial pathogens as well as phytopathogenic fungus species in significant amounts [34,35]. Vicilin has also been found to have considerable entomotoxic activity against seed stored insects [36,37]. The main objectives of this study were the isolation, purification and extensive functional characterization of *Vac*V from moth bean seeds, which correspond to antimicrobial and insecticidal proteins. The crude extract showed maximum concentration of *Vac*V (350 mg) and purification fold, which reduced in anion exchange (122.5 mg) and gel filtration (12.2 mg) chromatography with a purification fold of 2.85 and 28.6, respectively. This upward tendency is consistent with previous findings from other researchers [31,32,33]. Anion exchange column chromatography and size exclusion were used to purify *Vac*V to homogeneity. Purified *Vac*V exhibited a single major mother band of approximately 50 kDa under reduced and non-reduced conditions. However, 50 kDa vicilin protein has been characterized by LC-MS/MS: generated residual data exhibited 83 and 100% sequence identity with *V. unguiculata*, *V*. *radiata* trimeric vicilin, and *V. angularis*, respectively [37,38]. The *Vac*V of moth bean was found to have no intra or inter-chain disulfide linkages. Multiple sequence alignment of *Vac*V with vicilins of *Vigna angularis* (*Van*V), *Vigna radiata* (*Vra*V) and *Vigna unguiculata* (*Vun*V) shows α-helices, β-strands, Cupin I (54–206), Cupin II (259–419) and (N-A-T), with asparagine being the probable residue for linking of carbohydrate moieties. Since all four sequences are totally devoid of cysteine residues, inter or intra-chain disulfide linkages are absent. Some vicilins are glycosylated and have a single site for glycosylation (N/T-A-T). *Vac*V amino acid residues were searched on the NetNGlyc 1.0 Server (https://services.healthtech.dtu.dk/service.php?NetNGlyc-1.0 (accessed on 25 December 2021), which indicated the same residues. Asparagine is the probable amino acid for N-linked carbohydrate moieties, as already indicated by Fukuda et al., in *V*. *angularis* [35] and *P*. *vulgaris* [3]. It has been reported that a single glycosylation site (N-X-S/T) for *V. radiata* was observed, indicating the presence of mannose sugars inside the carbohydrate moiety [39]. Similarly, glycosylation (N/T-A-T) was also observed in *Vac*V. However, such glycans have no role in the folding and self-assembly of these vicilins. Thus, we have designed the computational three-dimensional model of *Vac*V-protein to gain the overview of 3D-organizations of amino acids. The model was of good quality with a high confidence score. Since then, the *Vac*V-protein structure has shared the most similarities with Chain A of the 7S Globulin-3 structure. For this reason, the structural superimposition showed the lowest RMSD value. This lowest RMSD value suggested the co-occurrence of *Vac*V and Globulin-3 proteins [40] (Figure 4A–E). It has been previously reported that vicilin possess several antimicrobial peptides [41]. In silico analysis of *Vac*V showed that those peptides have potent biological activities, including antibacterial, antifungal, ACE inhibition, antioxidant and insecticidal activities.

Phylogenetic analysis is a key to deciphering the evolutionary pattern of proteins [42]. Here, we were determined to analyze the evolutionary study of *Vac*V protein. Phylogenetic tree showed that *Vac*V protein formed the neighboring clade with *Vigna radiata*, which was quite obvious because they both belong to same protein family. It has been reported that the vicilin protein from *Vigna unguiculata* has two domains (bicupins), cupin_1 family (N-terminal) and cupin_2 family (C-terminal), which closely resemble *Vac*V protein domains [15]. All other amino acid sequences from other orders (*Brassicales*, *Caryophyllales*, *Ranunculales*, *Solanales*, and *Poales*) also have cupin family domains, indicating a close evolutionary relationship between them [16]. There was 100% vicilin protein similarity in *vigna aconitifolia* and *Vigna radiata*, which showed that there is evolutionary and structural sameness in the synthesis of the vicilin protein, which shares the same genes as the vicilin protein with the cupin superfamily. The N-terminus of vicilin is designed with 50–60 amino acid repeats containing antimicrobial activity motifs [43]. The vicilin protein contains several bioactive conserved peptides involved in antibacterial, antioxidant, antifungal, insecticidal and ACE-inhibitory function previously characterized [44,45,46,47,48,49]. Our results were consistent with Al Saiqali (Table S11), who reported antimicrobial and antioxidant activity peptides from *Azadirachta indica* leaves [50]. Microbial infections have acquired resistance to currently available antibiotics, prompting a surge in real interest in antimicrobial protein isolation [51]. Along with bacterial infections, plant pathogenic fungi have been documented to cause significant losses to agriculturally essential crops due to root degradation, resulting in significant economic losses worldwide [52]. The *V*. *aconitifolia* vicilin protein inhibits the growth of many pathogenic bacterial and fungal species, making it an ideal option for the development of novel antimicrobial drugs. The growth inhibition of bacterial species (*Escherichia coli*, *Pseudomonas aeruginosa* and *Xanthomonas campestris*) and fungal species (*Fusarium solani*, *Ustilago maydis*, *Alternaria helianthi*, *Fusarium oxysporum*, *Saccharomyces cerevisiae* and *Phytophthora cryptogea*) by vicilin has been reported previously [53,54,55]. The computational calculation of the *Vac*V inhibitory concentration responsible for 50% of the killing of the bacterial population was performed through a microtiter assay. In cupin domain families, conserved lysine (K), tyrosine (Y) and tryptophan (W) residues in the vicilin play a pivotal role in the chitin binding activity of bacterial strains chitinase. A variety of concentrations were used to challenge the bacterial pathogens, and a 20 μg/mL treatment was found to be efficient in inhibiting bacterial cell growth by more than 50%. *Capsicum annuum*, *Phyllostachys pubescent* and *Macadamia integrifolia* vicilin polypeptides strongly inhibited *B*. *subtilis*, *S*. *aureus*, *A*. *rhizogenes*, *E*. *coli*, *P*. *aeruginosa* and *X*. *campestris* growth at the concentrations of 15, 22, 50 and 100 μg, respectively [17,56]. Vicilin purified from cowpea and pea seed strongly inhibited the growth of bacterial species such as *Listeria monocytogenes*, *Listeria ivanovii*, *Streptococcus pyogenes*, *Klebsiella pneumonia*, *Pseudomonas aeruginosa*, *Bacillus lichniforms*, *Bacillus theriogensis* and *Salmonella* at a concentration of 10 to 200 μg [19,50]. Furthermore, the disc diffusion method was used to evaluate 20 μg *Vac*V concentration against Gram-negative (*P. aeruginosa*, *X. oryzae* and *E. coli*), which produced strong zones of inhibition against all strains in comparison to the negative control, and the least inhibition zone against Gram-positive bacterial species (*B. subtilis*, *S. aureus*)**.** In terms of antibacterial action, the vicilin protein differentiates Gram-negative bacteria from Gram-positive bacteria. These findings are similar to those of previous research [57,58]. The antibacterial activity of *Vac*V from *Vigna aconitifolia* seed could be linked to their high positive charges and, as a result, the hydrophobicity of such high molecular mass compounds, which facilitates electrostatic interactions with bacterial cellular components that damage cell integrity. As a result, the bacterial cells lose their ability to divide, resulting in emptied and destroyed cells [59,60]. Similarly, *Fusarium oxysporum* and *Aspergillus flavus* conidial germination and subsequent mycelial proliferation were significantly reduced by a 50 μg/well *Vac*V concentration. Vicilin extracted from *V*. *unguiculata* (20 mg; 800 μg), *Capsicum baccatum* (100–200 μg/mL), *Gossypium hirsutum* (60 μg), *Centrosema virginianum* (200–800 μg), *Cucumis melo* (50–250 μg) and *Lupinus angustifolius* (800 μg) inhibited growth of *Saccharomyces cerevisae*, *Fusarium solani*, *Fusarium oxysporum*, *Candida tropicalis*, *Collectotricum musae*, *Phytophthora caprici*, *Neurospora crassa*, *Ustilago maydissporidia*, *Botrytis cinerea*, *Candida albicans*, *Candida tropicalis*, *Kluyveromyces marxiannus*, *Sclerotinia sclerotiorum* and *Phytophthora nicotianae* [54,61]. To validate our results, a 50 μg *Vac*V concentration was tested on two fungi in Petri dishes, and considerable inhibition of fungal mycelia was observed as compared to a negative control. An assessment of reported vicilin concentrations revealed that *Vac*V has a high potency against fungal pathogens and is a promising target for therapeutic development. Previous findings suggest that vicilins are associated with fungal cell walls, possibly due to the chitin component in these structures [62,63]. Since chitin-binding proteins (vicilin) contain abundant positively charged residues (such as arginine), they could interact with the negatively charged components of the cell membrane, causing membrane disruption (depolarization of lipid membranes) and cell lysis (Figure S4) [64]. It is generally known that chitin found in fungal cell walls interacts with a group of proteins known as chitin binding proteins [20,53], causing growth inhibition in these species, as shown in [44,65]. These findings suggest that legume seed vicilins may interact with organisms that contain glucose, sucrose, chitin, chitin derivatives, or *N*-acetylglucosamine-containing glycoconjugates in structures exposed to the outside environment [66]. Some proteins, such as vicilins, which have shown chitin binding affinity, which has been attributed to their insect toxicity [67]. It has been previously reported that vicilin extracted from a variety of legumes and plant seeds, including *Phaseolus vulgaris*, *Phaseolus lunatus* [22], *Vigna angularis* [35], *Canavalia ensiformis* [16], *Glycine max* [68], *Vigna unguiculata* [69], *Enterolobium contortisiliquum* [53], *Anadenanthera colubrina* [53] and *Albizia lebbeck* [63], showed insecticidal activity against *Callosobruchus maculatus* [70], *Diatraea saccharalis* [69], *Tenebrio molitor* [71], *Plodia interpunctella* [64], *Ceratitis capitate* [64] and *Zabrotes subfasciatus* [53], with a significant decrease in larval development and adult growth. Similarly, *Vac*V showed strong entomotoxic activity against the most harmful stored grains insects, *S. oryzae*. When *S. oryzae* larvae and pupae were exposed to *Vac*V mixed flour, the population of larvae and pupae was greatly reduced, and the larvae and pupae did not complete metamorphosis into adults. The maximum concentration of *Vac*V (3.0 mg/20 g of rice kernels) showed maximum mortality rate of *S. oryzae* larvae and significantly decreased in F1 generation adults (35 and 65 days), along with adult males and females in the F2 generation. Vicilins have insecticidal properties because they link to chitinous structures in midguts of insects, preventing them from development [72]. Moth bean vicilin, for example is bound with chitin, a polysaccharide of β-1,4 connected with GlcNAc units found in the exoskeleton of insects, crustaceans, and other invertebrates. These findings are consistent with earlier research on other stored grain insect pests [26]. Since the *Vac*V protein inhibits the growth of many pathogenic bacterial and fungal species, it is an ideal candidate for the development of novel antimicrobial drugs [73]. Plant vicilin is cytotoxic and has been demonstrated to have anticancer properties [50]. Moth bean seed extract (*Vac*V) was tested for antiproliferative efficacy and cytotoxicity in HepG-2 liver cancer cells. Different concentrations of *Vac*V were used, but 180 μg per well significantly killed cancerous cells (70%). This research used the protein caspase-3, receptor was taken from the Protein Data Bank (PDB) and used as a target for stimulating docking against the *Vac*V protein. These findings suggest that interactions between the *Vac*V and caspase 3, a protein found in human cancer cell lines, could be significant (Table S10). Furthermore, it is well known that recognizing the ligand-receptor binding site is the starting point for drug development as well as determining the function of a protein. Gupta et al. has revealed that mung bean vicilin protein hydrolysate has cytotoxic effect against MDA-MB-231 and MCF-7 breast cancer cell lines. They also looked at the effect of vicilin peptides on angiogenesis converting enzyme (ACE) inhibition [73]. Herein, vicilin could be a promising candidate for anticancer medication development and formulation. The structural knowledge of vicilin proteins can be used to develop novel anticancer and antimicrobial drugs. 

Although we have characterized the *Vac*V protein, there is still plenty of room available to further validate its potential therapeutic activity in plants and humans. There are some limitations associated with this study. For example, there is a lack of anticancerous and antiviral activity validation in human cell lines and plants, respectively. So, combining all these results, we unveil the functional and structural characterization of the *Vac*V protein. Thus, this functional characterization of moth bean seed vicilin may convince the hungry population to adopt the crop as part of their routine diet as an alternative to more expensive food crops. The exploration and characterization of such molecules may contribute towards the control of human diseases and plant pathogens.

## 4. Materials and Methods

### 4.1. Biological Materials and Growth Conditions

*V. aconitifolia* seeds were obtained from the Botanical Garden, Bahauddin Zakariya University, Multan, Pakistan and stored at room temperature. *S*. *aureus*, *E*. *coli*, *B*. *subtilis*, *P*. *aeruginosa* and *X*. *oryzae* bacterial strains used in this study were purchased by the Institute of Pure and Applied Biology (IP&AB), Faculty of Microbiology, Bahauddin Zakariya University, Multan, Pakistan. *F*. *oxysporum* (FCBP-PTF-866) and *A*. *flavus* (FCBP-PTF-862) were acquired from First Culture Bank of Pakistan (FCBP) Lahore, Pakistan. *S*. *oryzae* (rice weevil) was provided by the Department of Entomology, Bahauddin Zakariya University, Multan, Pakistan. Hepatoma liver cell lines (HepG-2) and assay related chemicals were kindly provided by the Centre for Applied Molecular Biology (CAMB), Punjab University, Lahore, Pakistan. All chemicals used in experiments were of analytical grade. 

### 4.2. Isolation and Purification of VacV

*Vac*V was isolated from *V. aconitifolia* seeds according to the procedure of Laemmli with some modifications [74]. Briefly, seeds (10.0 g) of *V. aconitifolia* were ground by pestle and mortar in liquid nitrogen and homogenized in 100 mL of phosphate-buffered saline (PBS, 100 mM, pH 7.5). The powder was dispensed in the beaker and stirred for 3–4 h and centrifuged at (13,000 rpm for 10 min at 4 °C). The resultant pellet contains plant debris, which was discarded, and the supernatant was collected and filtered (filter paper, 0.8 µm). The resulting crude extract was precipitated with saturated ammonium sulfate (60%) at 4 °C. After centrifugation at (3000 rpm for 3 min at 4 °C), the supernatant was collected, and the obtained pellet was discarded. The salt was removed from the resultant supernatant through the dialysis membrane of 3.5 kDa MWCO (Spectra/Por 3; Catalog no. 132724) in the same buffer. The dialyzed partially pure sample was loaded on to the Hi-Load 16/60 Superdex 200 pg. column using extraction buffer (PBS at a flow rate of 0.5 mL/min). UV detector (280 nm) was used for the recording of eluent absorbance. Column calibration was performed with a set of known molecular weight proteins: alcohol dehydrogenase (150 kDa), ovalbumin (42.7 kDa), lysozyme (14.4 kDa) and proteinase K (28.9 kDa). After SDS-PAGE analysis, the fractions with maximum purity were pooled and subjected to an anion exchange column (Hi trap Q FF column). The column was equilibrated with 5 mL of PBS followed by the injection of a 1 mL *Vac*V sample was injected into the column. The *Vac*V was eluted in 0.5 mL fractions using a linear NaCl gradient (0–500 mM). Fractions with maximum purification and quantities were pooled together and loaded on SDS-PAGE under reduced and non-reduced conditions.

### 4.3. Characterization of the Purified Protein

#### LC-MS/MS Mass Spectrometry Analysis

Protein quantification was performed by the Bradford method by using a standard of Bovine Serum Albumin (BSA) [75]. SDS-PAGE (12%) as a standard protocol was used to analyze the protein banding patterns. The molecular weight of the protein was determined by comparing electrophoretic mobility with a protein marker (Bioscience). The gels were treated with 0.25% (*w*/*v*) Coomassie Brilliant Blue R-250 (CBB R-250) in methanol/water/acetic acid (50:40:10) for staining and with the same solvent without Coomassie dye for destaining [76]. Profiling and identification of proteins and peptides were performed using the Nano liquid chromatography system (Dionex UltiMate 3000) and orbitrap fusion mass spectrometry (Orbitrap Fusion, Germany). Briefly, the stained protein bands were excised from the gel and reduced with dithiothreitol (5 mM, at 55 °C and for 30 min). The protein was digested with trypsin (5 ng/µL; sequencing grade trypsin, Promega, Madison, WI 53711-5399 USA) in 50 mM NH_4_HCO_3_ at 37 °C for 16 h. The acetonitrile (50%) or 5% formic acid solution was used to extract the digested gel pieces, which were dried in a vacuum concentrator. The peptide mixtures were separated on a nano-high liquid chromatography system (Dionex UltiMate 3000), and nano-HPLC was coupled via electrospray-ionization to an orbitrap mass spectrometer (Orbitrap Fusion, Germany). LC-MS/MS measurements were analyzed in data-dependent acquisition mode (DDA). The thermo proteome Discoverer 2.0 (Thermo Scientific, Germany) was used to further process the LC-MS/MS raw data. The MS data was investigated with Sequent HT against the *Arabidopsis* and the UniProtKB server, while identifications were validated manually.

### 4.4. Bioinformatics Analysis

#### 4.4.1. Multiple Sequence Alignment and Evolutionary Assessment

In order to find the amino acid conservation of *Vac*V protein among different plant species (*V. radiata*, *V. angularis* and *V. unguiculata*), we performed multiple sequence alignment (MSA) by using Clustal Omega [77]. Briefly, alignments were performed on sequences from the same species as well as sequences from different species. The BLOSUM (Block Substitution Matrix)-type substitution/identity matrices [78] were calculated for this purpose with the help of the BioEdit software tool. To analyze evolutionary relationships, vicilin consensus sequences were combined with sequences from other species. The NJ (Neighbour Joining) method [79] with 1000 bootstrap value was used to create phylogenetic trees based on the BLOSUM-type matrix using the amino acid sequence alignment generated by ClustalW. The Treedyn software application was used to visualize the trees [78]. 

#### 4.4.2. Molecular Modelling and Docking

*Vac*V protein structure was predicted by applying the homology modelling technique according to Eswar and coworkers [80]. Modeller V9.14 was used for structure prediction at default parameters and also verified by I-TASSER server https://zhanglab.ccmb.med.umich.edu/I-TASSER (accessed on 27 February 2022). The chain A of 7S Globulin-3 (PDB: 2EAA_A) was selected as a best template with a homology of 94% and 0.0 E-value. The best output model was selected on the basis of its lowest energy and highest RMSD value. Structural energy minimization was performed by UCSF Chimera at default settings [81]. The Molprobity server [82], ProSA-web [83], and PROCHECK platform [84] were used to evaluate model quality. Structural analysis was performed by EBI-PDBsum and visualized through UCSF Chimera [85]. In silico protein–protein interaction analysis was performed between *Vac*V protein model and Caspase3 (PDB ID: 3DEI) using HDOCK-Server [http://hdock.phys.hust.edu.cn/] (accessed on 27 February 2022) to explore the mode of interactions. HDOCK uses integrated approach in two modes, i.e., hybrid docking mode (Default mode), and the other is template-free docking mode [86]. Here, we have applied default mode, pdb format of *Vac*V receptor and 3DEI ligand were used as an input, lowest energy (–247.44 KJ/mol) ranked complex was selected as a best docked conformation. Three-dimensional complex was visualized in ribbon form using UCSF-Chimera.

### 4.5. Biological Assays

#### 4.5.1. Antibacterial Activity

The antibacterial bioassay against two Gram-positive pathogenic bacterial species, i.e., *B. subtilis*, *S. aureus*, and three Gram-negative pathogenic bacterial species, *E. coli*, *P. aeruginosa*, and *X. oryzae*, was performed using the disk diffusion method according to a previously described protocol [87]. Briefly, all bacterial strains were cultured in Luria-Bertani (LB) medium and incubated at 37 °C until the optical density (OD) reached 0.1 absorbance unit. An amount of 100 μL of fresh bacterial culture was treated with 100 μL of *Vac*V containing different concentrations (10, 15, 20, 25, and 30 μg). Positive and negative controls were bacterial cells treated with antibiotics (Amoxicillin (1 mg mL^−1^)) and PBS, respectively. Five replicates of all the treatments were made and absorbance was recorded at 600 nm on an hourly basis up to 8 h of incubation at 37 °C using the standard protocol of Kirby–Bauer susceptibility test against pathogenic bacteria [88]. After careful observation of the microbial growth inhibition as a result of different *Vac*V concentrations, two treatments (15 and 20 μg) were further assessed. The experiment was performed in triplicate and zones of inhibition were recorded for every individual replicate and mean values were calculated.

#### 4.5.2. Antifungal Assessment

The antifungal activity of *Vac*V was tested against two pathogenic strains of fungi *F. oxysporum* (FCBP-PTF-866) and *A. flavus* (FCBP-PTF-862) according to Balouiri [89]. The cultures were kept at 4 °C and proliferated on starch agar peptone yeast media (YPSA). Mature conidia of both fungi were prepared and collected from Petri dishes by using 10 mL of sterilized PBS buffer (pH 7.5). Asexual spores were counted at 400× magnification in a binocular microscope (Ernst Leitz Wetzlar GMBH, Germany) by using a hemocytometer (NeubauerHausser Bright-Line; Catalog No. 3100) and adjusted to a standard concentration of 1 × 10^5^ cell mL^−1^. Conidial germination and mycelial growth inhibition were tested against different molar concentrations of *Vac*V (30, 40, 50, 60 and 70 μg/100 μL/well) by using the 96 well microtiter plate in 200 μL volume at 25 °C. PBS was used as a negative control, while the fungicide Topsin^®^ 4.5 FL (10 μL) was used as a positive control. The absorbance was recorded at 600 nm with periodic time intervals of 0, 24 and 48 h of post-incubation. Statistical values including means, standard errors and coefficients of variation were calculated and plotted to see the fungal growth patterns. Further, 50 μg/disc *Vac*V concentration was reevaluated against the two fungi by using a disc diffusion assay similar to an antibacterial experiment. The sterilized discs (15 mm) of filter paper were placed at equidistance from the center of the Petri plate. Fungicide Topsin^®^ 4.5 FL (10 μL) was taken as a positive control and PBS (60 μL) was taken as a negative control, while 50 μg/disc *Vac*V was used for fungal growth inhibition. The fungus culture was placed carefully in the center of the Petri plate and kept in an incubator at 30 °C. After 48 h of incubation, the fungal growth was analyzed, and the results were recorded. 

#### 4.5.3. Determination of Insecticidal Activity

Insecticidal potential of *Vac*V was evaluated against the stored rice pest *Sitophilus oryzae*. Techniques to maintain insect cultures were adopted as per standard protocol [26]. All experiments were conducted for 35 days in a controlled environment chamber at 27 °C and 70% relative humidity. In first batch, 100 g of broken rice kernels were sprayed with different concentrations of *Vac*V (1.0, 2.0 and 3.0 mg) and a control (PBS 100 mM, pH 7.0) to test the effect of protein on the insect population. Five replicates (20 g kernels in each replicate) were examined, and 20 male and female insects were fed on this diet in a glass jar in each replicate. Survival data was noted after every 3 days up to 9 days and number of surviving adults were noted and compared with control [26] In the second batch, the rice kernels having the progeny of adults were taken and hatched at an optimum temperature of 27 °C for 35 days of incubation time with the same contaminated rice kernels. The surviving individuals were left for the next progeny to see the protein effectivity on the lifespan of rice weevils after 35 (F1 generation) and 65 (F2 generation) days [65]. 

#### 4.5.4. Assessment of Anticancer Activity toward HepG-2 Cancerous Cell Lines

*Vac*V antiproliferative activity was determined by using human hepatoma (liver) cells as previously described [73,90] Briefly, The HepG-2 cell line was cultivated and maintained at 37 °C in a 5% CO_2_ atmosphere in Dulbecco’s modified Eagle medium (DMEM) containing 100 U/mL penicillin, 100 g/mL streptomycin, and 10% Fetal Bovine Serum (FBS). The MTT test kit (Millipore, Burlington, MA, USA) was used to measure cell survival and proliferation. *Vac*V protein was diluted in a range of concentrations (10 to 180 μg/mL, 3 wells per concentration) and incubated at 37 °C in a humidified CO_2_ incubator. After incubation, triplicates of *Vac*V concentrations ranging from 10 to 180 μg/mL were added to the wells of the plate, and the plate was incubated at 37 °C for 24 h in a CO_2_ incubator. After incubation, the exhausted media was removed, and new media was added to each well, along with MTT reagent (5 mg/mL in PBS) according to the manufacturer’s instructions. The plate was then incubated for 4 h at 37 ° C in a CO_2_ incubator. DMSO (100 microliters) was added to the wells to dissolve the formazen crystals that formed following incubation. The optical density of the contents of the plate was measured using an ELISA plate reader as indicated in the technique to quantify the MTT formazen product. Cells treated with 0.1% DMSO were used as a solvent control, whereas untreated cells were used as an OD control.

The following formula was used to obtain cell viability.

(Viable cell) % = (OD (570–620 nm) of drug treated sample/OD (570–620 nm) of untreated sample) × 100.

## 5. Conclusions

In this study, vicilin protein identification, purification, structure analysis and functional characterization have been described briefly. The practice concedes for large-scale protein preparation suitable for nutritious food as an alternate to more expensive food crops. Bioinformatics analysis, including protein structure and phylogenetic analysis, which provides a framework for further study of vicilin protein in *vigna aconitifolia* and other *Fabaceae* species. In addition, comprehensive functional analysis of *Vac*V against bacteria, fungi, insects and cancerous cell line shows significant effects, indicating that vicilin has antimicrobial potency. Finally, all the data herein can lead to new explorations of the functional, nutritional and medicinal properties of vicilin protein in moth beans. However, the whole mechanism (in vitro study) underlying the therapeutic potential of the moth bean seed protein must be thoroughly studied.

## Figures and Tables

**Table 1:** Purification steps of *V. aconitifolia* vicilin (*Vac*V) from one gram of seed powder.

Purification Steps	Total Protein (mg)	Purification (times)	Recovery Yield (%)
Crude	350	1	100
Ammonium sulfate fractionation (60%)	245	1.42	70
Hi-Load 16/60 Superdex	122.5	2.85	35
Hi trap Q FF C	12.2	28.6	3.48

Notes: mg: milligram; %: percentage.

**Table 2:** LC-MS/MS generated amino acid sequences of *Vigna aconitifolia* Vicilin (*Vac*V).

Sr. No.	Peptide Sequences (25 kDa)	
1	GKNNPFYFNSDR ^(12)^	
2	QMQNLENYR ^(09)^	
3	AVLTLVNPDGGR ^(12)^	
4	IPAGTIFFLVNPDDNENLRIIKLAVPVNNPHRFQDFFLSSTEAQQSYLQGFSKNILEASFDSDIKEINRVLFGEEGQQQQQGQESQQEGVIVELKR ^(96)^	
5	SLSSEDQPFN ^(10)^	
6	DLDVFISSVDMKEGSLLLPHYNSKAIVILVINEGEANIELVGL ^(43)^	
7	NFLAGEKDNVISEIPTEVLDLAFPAPGEKVEK ^(32)^	
**Sr. No.**	**Peptide Sequences (30 kDa)**	**Peptide Sequences (40 kDa)**
1	LSYFVDAQPQQK ^(12)^	TVSSEDEPFNLR ^(12)^
2	VLEVAFPGSVSK ^(12)^	NPAGTLFFLVNPDDNENLR ^(19)^
3	TVSSQNEPFNLR ^(12)^	FQDFFLSSTEAQQSYLQGFSK ^(21)^
**Sr. No.**	**Peptide Sequences (50 kDa)**	
1	GKNNPFYFNSDR ^(12)^	
2	SKQMQNLENYR ^(11)^	
3	AVLTLVNPDGGR ^(12)^	
4	AGTIFFLVNPDDNENLRIIKLAVPVNNPHRFQDFFLSSTEAQQSYLQGFSKNILEASFDASDIKEINRVLFGEEGQQQQQGQESQQEGVIVELKR ^(95)^	
5	SSSKKSLSSEDQPFNLR ^(17)^	
6	NPQLKDLDVFISSVDMKEGSLLLPHYNSKAIVILVINEGEANIELVGLR ^(49)^	
7	FGINAENNQRNFLAGEKDNVISEIPTEVLDLAFPAPGEKVEK ^(42)^	

Notes: kDa: kilo Dalton; numbers (12), (09), (10), (43), (32), (19), (21), (11), (96), (95), (17), (49) and (42) total amino acids in each fragment

**Table 3:** Protein identification was performed by using the amino acid sequence of first fragment obtained from LC–MS/MS.

Plants	Sequence	Protein Name	Homology (%)	Accession No.
*V. aconitifolia*	GKNNPFYFNSDR	*Vac*V	100	This Study
*V. radiata*	GKNNPFYFNSDR	8S*α*	100	A0A3P9QP39
*V. angularis*	GKNNPFYFNSDR	*Van*V	100	A0A0L9U0Y5
*V. unguiculata*	GQNNPFYFDSDR	Vicilin	83.3	A8YQH5

Notes: *Vac*V means *Vigna aconitifolia* vicilin, 8S*α*; 8S globulin alpha isoform and *Van*V; *Vigna angularis*.

**Table 4:** Antibacterial activity of purified *Vac*V at different concentrations and positive control (Calamox, Bosch) against different pathogenic bacteria.

Protein	Bacteria	Inhibition Zone Diameter (mm)		
		*Vac*V 15 μg/disc	*Vac*V 30 μg/disc	Antibiotic 5 μg/disc
*Vac*V	*Bacillus subtilis*	15 ± 2	18 ± 2	28 ± 1
*Vac*V	*Escherichia coli*	13 ± 2	17 ± 2	27 ± 2
*Vac*V	*Pseudomonas aeruginosa*	16 ± 2	20 ± 3	30 ± 1
*Vac*V	*Staphylococcus aureus*	11 ± 2	16 ± 2	22 ± 2
*Vac*V	*Xanthomonas oryzae*	18 ± 2	21 ± 1	31 ± 1

Notes: mm: millimeter; μg/disc: microgram per disc; *Vac*V: *Vigna aconitifolia* vicilin.

## Data Availability

All data generated or analyzed during this study are included in this published article. The data presented in this study are available on request from the authors.

## Supplementary Materials

The following supporting information can be downloaded at: https://www.mdpi.com/article/10.3390/molecules27103251/s1, Table S1: Different percentage of Ramachandran plot; Table S2: Position and types of gamma turns; Table S3: Beta hairpins in *Vac*V strands with amino acid position and length; Table S4: Number of beta bulges with different types in *Vac*V; Table S5: Number of strands and types of beta sheets; Table S6: Number of helices and sequence of amino acids; Table S7: Number of helices and sequence of amino acids; Table S8: Confirmation of H-bond and turn type in beta turns; Table S9: Amino Acid Conservation Scores; Table S10: Docking Score—247.47; Table S11: Bioactive peptides of vicilin with different activities in different species. Figure S1: *Vac*V purification followed by chromatography; Figure S2: Two-dimensional topology of *Vac*V protein; Figure S3: The 96-well micro plate used in MTT assay; Figure S4: Mode of action of *Vac*V against bacteria, fungi and insect.

## Sample Availability

Samples of the compounds are not available from the authors.
