# Supplementary material for: In Silico Analysis and Functional Characterization of Antimicrobial and Insecticidal Vicilin from Moth Bean (Vigna aconitifolia (Jacq.) Marechal) Seeds
Source: Molecules. 2022 May 19;27(10):3251. doi: 10.3390/molecules27103251 (PMC9145308; doi:10.3390/molecules27103251)
Supplement: Supplementary file 1 [file molecules-27-03251-s001.zip › Supplementary_Figure-4.24.pdf]

Supplementary Figures:

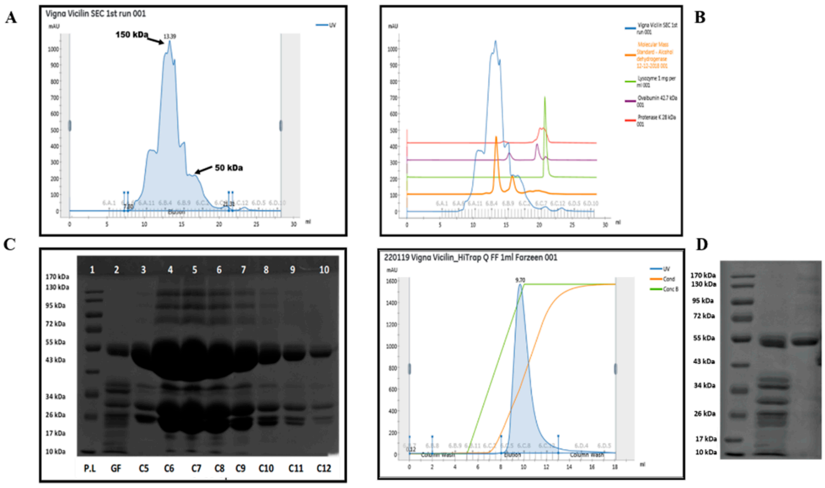

Figure S1: *Vac*V purification followed by chromatography. (**A**) Gel filtration (Hi-Load 16/60 Superdex 200 pg column) chromatogram of *Vac*V after ammonium sulfate precipitation. A very typical chromatogram showing the presence of 150 kDa trimeric and 50 kDa monomer of the protein. (**B**) Gel filtration chromatogram of different standard proteins for the calibration and calculations of molecular weights. (**C**) Anion exchanger chromatogram of the selected 150 kDa fractions eluting from gel filtration. Very highly purified *Vac*V fractions were pooled together and ran on the SDS-PAGE under reduced conditions. (**D**) *Vac*V banding pattern corresponding to the highly purified fractions (C5 to C12) of QFF exchanger column. Fractions were pooled together and were used for further characterization. GF is the banding pattern of gel filtration fractions and P.L is the standard protein ladder (Catalog no. 26616).

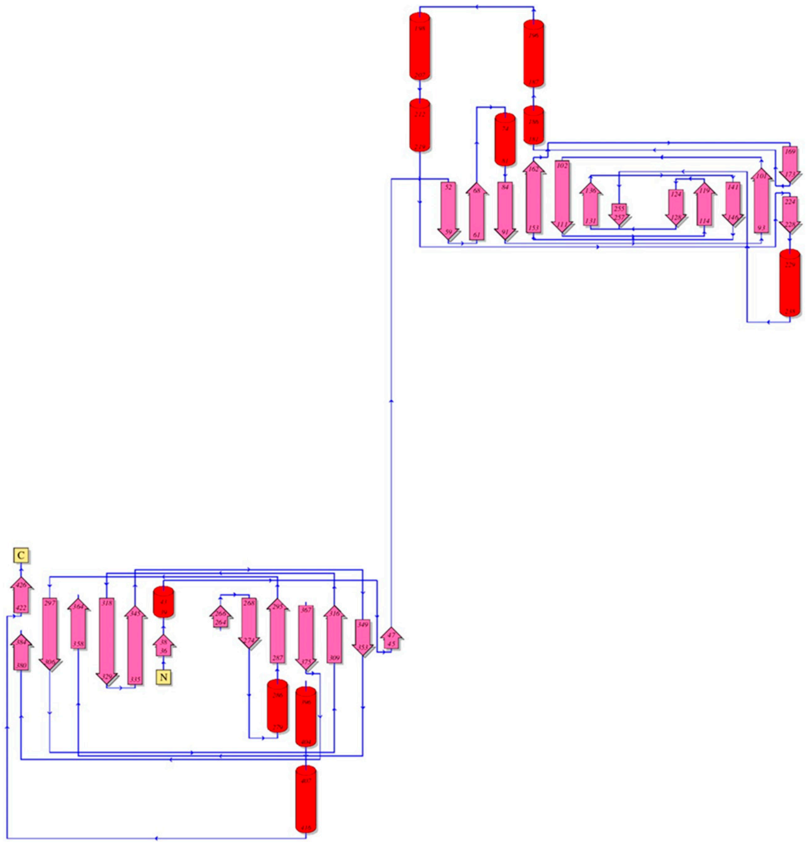

Figure S2. Two-dimensional topology of *Vac*V protein. Red barrels are showing the position of helices, pink arrows are interpreting the direction and position of sheets and blue lines are showing connecting loops.

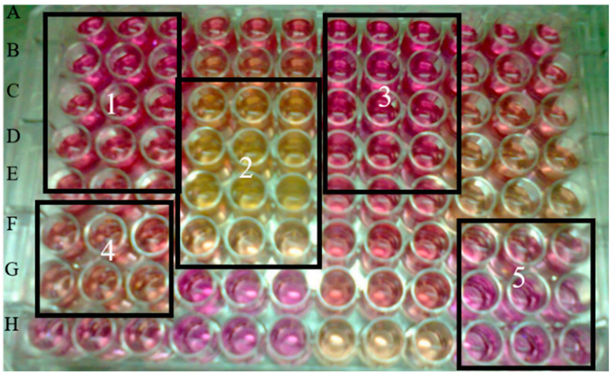

Figure S3. The 96-well micro plate used in MTT assay. (1) Untreated cells control wells, (2) cytotoxicity with 180 µg /well *Vac*V vicilin, (3) cell lines with 120 µg/well of *Vac*V, (4) cell lines with 120 µg/well of *Vac*V, (5) cell lines with 90 µg/well of *Vac*V.

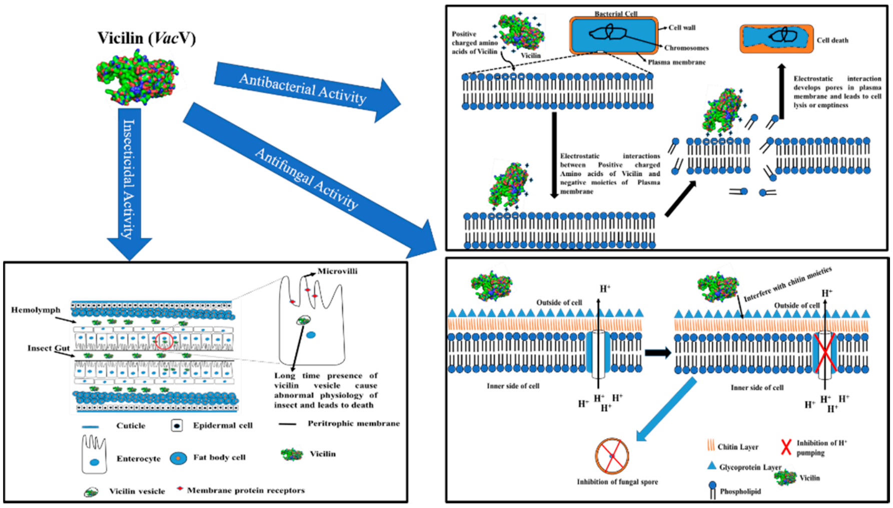

Figure S4. Mode of action of *Vac*V against bacteria, fungi and insect.
